# Think and Choose! The Dual Impact of Label Information and Consumer Attitudes on the Choice of a Plant-Based Analog

**DOI:** 10.3390/foods13142269

**Published:** 2024-07-18

**Authors:** Elson Rogerio Tavares Filho, Ramon Silva, Pedro Henrique Campelo, Vitor Henrique Cazarini Bueno Platz, Eduardo Eugênio Spers, Mônica Queiroz Freitas, Adriano G. Cruz

**Affiliations:** 1 Department of Food Technology, Universidade Federal Fluminense (UFF), Niterói 24230-340, RJ, Brazil; mqfporto@gmail.com; 2 Instituto Federal do Rio de Janeiro (IFRJ), Rio de Janeiro, Rio de Janeiro 20270-021, RJ, Brazil; ramonsg@uol.com.br; 3 Department of Food Technology, Universidade Federal de Viçosa, Viçosa 13081-970, MG, Brazil; pcampelo.felix@gmail.com; 4 Escola Superior de Agricultura Luiz de Queiroz (ESALQ), Universidade de São Paulo (USP), Piracicaba 13418-900, SP, Brazil; vitorhenriqueplatz@usp.br (V.H.C.B.P.); edespers@usp.br (E.E.S.)

**Keywords:** plant-based, consumer choice, attitudinal factors, stated preference, non-dairy

## Abstract

This study explored the impact of various label information (extrinsic attributes) and sociodemographic and attitudinal factors (intrinsic attributes) on Brazilian consumer choices, using simulated traditional and plant-based muçarela cheese as the model product. The research was conducted in two phases: the first involved a structured questionnaire assessing attitudinal dimensions such as Health Consciousness, Climate Change, Plant-based Diets, and Food Neophobia, along with sociodemographic data collection. The second phase comprised a discrete choice experiment with (n = 52) and without (n = 509) eye tracking. The term “Cheese” on labels increased choice probability by 7.6% in a general survey and 15.1% in an eye tracking study. A prolonged gaze at “Cheese” did not affect choice, while more views of “Plant-based product” slightly raised choice likelihood by 2.5%. Repeatedly revisiting these terms reduced the choice probability by 3.7% for “Cheese” and 1% for “Plant-based product”. Nutritional claims like “Source of Vitamins B6 and B12” and “Source of Proteins and Calcium” boosted choice probabilities by 4.97% and 5.69% in the general and 8.4% and 6.9% in the eye-tracking experiment, respectively. Conversely, front-of-package labeling indicating high undesirable nutrient content decreased choice by 13% for magnifying presentations and 15.6% for text. In a plant-based subsample, higher environmental concerns and openness to plant-based diets increased choice probabilities by 5.31% and 5.1%, respectively. These results highlight the complex dynamics between label information, consumer understanding, and decision-making.

## 1. Introduction

The rise of plant-based dairy analogues has been driven by changes in dietary habits and shifts in governmental and industrial chains towards sustainable development goals, which notably include zero hunger, sustainable agriculture, and combating climate change [1]. In this scenario, plant-based substitutes offer sustainable solutions for population growth and the uneven distribution of food, particularly in regions with limited dairy production due to climatic, geographic, or economic constraints [2].

Concerns about the environmental impact of intensive agriculture, which consumes substantial resources and emits greenhouse gases, motivate the search for more sustainable and environmentally friendly plant-based alternatives and analogues. Additionally, high inflation and rising production costs in the dairy sector drive the search for more economical options.

Various plant-based alternatives, such as soy, almonds, oats, rice, and coconut, are used to create dairy analogues [3]. Soy, historically introduced in Brazil as an alternative to animal-based products, faced consumer resistance due to the negative perception of many consumers towards the bean and its derived products [4]. More recently, cashew-based analogs have gained popularity in Brazil [5]. Among other reasons that may be associated with a greater openness to analogues made with cashew nuts, market analysis by Craig et al. [6] observed that cashew-based products commonly present higher levels of protein and lower levels of sodium and saturated fat than those based on cow’s milk or coconut.

Despite the recognized advantages of plant-based alternatives, there are still gaps in understanding how consumers perceive such products, whether in terms of composition [7], nomenclature (e.g., vegetarian, vegan, or plant-based product), and the claims (e.g., sustainability, health, animal welfare) used to designate these products and their benefits [8,9,10]. Therefore, it is crucial to increase our understanding of the factors that influence consumer choices and how label information affects these decisions [11,12]. In Brazil, as in many other countries, legislation establishes specific guidelines for plant-based products [13]. Although the feasibility and advantages of producing plant-based dairy alternatives are well recognized, there is still a significant gap in understanding how these products are perceived by consumers and, consequently, how label information and related legal requirements impact consumers at the point of purchase.

An emerging issue in Brazil concerns the creation of definitive legislation to establish the nomenclature and quality standards of plant-based dairy analogues. Currently, in line with other international legislation [14], it is prohibited in Brazil for plant-based analogs to be called or classified in the same category as their animal-derived counterparts [13]. This means that plant-based analogues cannot be marketed or labeled as cheese, yogurt, or butter, terms that are already familiar and established among consumers. Although the legislation aims to protect consumers and prevent confusion, it is observed that many consumers continue to use nomenclatures associated with animal products to describe plant-based products. This occurs even though they are aware that they are made from plants, as noted in the market research by the International Food Information Council [15]. In the survey, which investigated the attitudes of just over 1000 consumers regarding the labeling of dairy products and non-dairy alternatives, it was observed that most consumers understand which products contain cow’s milk and which do not, regardless of whether they are labeled with a dairy-related term. For example, a large part of the respondents recognized the use of the name milk in plant-based alternatives without confusing it with animal milk, with fewer than 10% of respondents believing that coconut, soy, cashew, and rice “milk” contained cow’s milk. Additionally, Brazilian legislation, although not definitive, has guided the declaration of the main ingredient of the plant-based product in a prominent way on the label, aiming to assist consumers in better understanding the composition of the products they choose. The effect of this type of declaration has also been poorly studied.

Regarding the labeling of food products, a mandatory front-of-package labeling (FoPL) system was recently introduced in Brazil, structured in the form of a magnifying glass associated with declarations of high levels of nutrients that contribute to non-communicable chronic diseases, such as sugar, sodium, and saturated fat [16]. This initiative aligns with global efforts to improve nutritional transparency, using clear symbols like a magnifying glass to alert consumers. Although this approach is promising, the efficacy of these labels in influencing consumer decisions requires additional studies, especially regarding the magnifying glass system, which is mandatory only in Brazil and still optional in Canada. International health agencies, such as the Pan American Health Organization [17], recommend the use of warning images on front-of-package labeling systems (FOPL), aiming to promote more effective communication of the risks associated with the consumption of certain nutrients. These pictorial structures are designed to convey an immediate alert message. Classic examples include the use of octagons, similar to “stop” traffic signs, or traffic light systems that use the color red to indicate high levels of harmful nutrients. These symbols are intentionally designed to attract attention and convey a message of caution or prohibition.

However, the system based on the magnifying glass does not align entirely with PAHO’s suggestions, as the magnifying glass, by its nature, may not be as incisive in conveying the idea of alert since it is more frequently linked to the idea of inspection or detailed observation, which may not evoke the same urgency or caution as traditional warning symbols.

In this context, discrete choice experiments (DCEs) have proven to be a useful tool for evaluating the decision-making process of consumers when faced with food products containing different information and claims [18]. DCEs are based on random utility theory [19], which assumes that consumers inspect products for information and weigh it to find the alternative with the highest utility, following a compensatory decision rule [20]. This allows researchers to decompose individual preferences through their choices, inferring how a particular attribute (expressed in its levels) relates to the choice, even if the consumer is not fully aware of its value, relating to subjective factors, heuristics, and low-involvement decisions [18]. However, slight deviations from the RUM assumption are expected, as consumers may decide without processing all the provided information, considering only part of it (non-compensatory decision rules) [21]. These deviations are expected, given the established relationship between bottom-up and top-down factors in the decision-making process. While bottom-up factors involve all the visual information provided by the product, whether on its label, packaging, or constitution, and can be objectively accessed by the senses, top-down factors involve the consumer’s intrinsic mental construct, including the willingness to process information, subjective beliefs, and attitudes [22].

To capture information related to top-down factors, studies using DCEs often include data associated with subjective beliefs, such as those obtained through attitudinal scales [23]. However, unread and unprocessed information may still escape top-down processing. In this context, eye tracking technology has shown great potential for simultaneously collecting data on information search and inspection processes as well as consumer preferences. Eye tracking allows for the monitoring and measurement of consumers’ eye movement trajectories, providing insights into how products are viewed and inspected. Additionally, the relationship between visual inspection, judgment, and decision-making (in this case, choice) supports the use of eye tracking in DCEs [22]. This occurs because visual attention in the perception process involves directing the open visual field (more general and diffuse) to a specific point (stimulus), transferring the visual stimulus to the foveal region of the retina. The fovea is known for its high density of sensory neurons and greater capacity for specialized visual processing [24]. The eye activity metrics most considered in decision-making processes are related to oculomotor activity [25], usually involving measures associated with fixation (the eye remaining on a specific point) and measures related to saccades (visits and revisits to a specific point), to a lesser extent blinks, and changes in phasic pupil diameter. In the context of choices, visits and revisits have been used to understand how individuals seek information to make a decision, and fixation has been used as an indicator of attention direction to information with the potential to influence the decision.

This study aimed to investigate how consumer choice is influenced by information required by identity legislation for analogous products commonly found on cheese and plant-based cheese labels. It also examined how consumers visually inspect this information. Specifically, the study focused on how the choice process is affected by product nomenclature (‘Cheese’ vs. ’Plant-based product’), main ingredient declaration (‘Made with milk’ vs. ‘Made with cashew nuts’), nutritional benefit claims (‘Source of vitamins B6 and B12’ vs. ‘Source of proteins and calcium’), and different front-of-package labeling structures (high saturated fat and sodium information presented with a magnifying glass image, as required by Brazilian legislation, and in simple text form). Additionally, the study assessed whether sociodemographic characteristics, consumption habits, and attitudes towards plant-based products (neophobia, health consciousness, environmental concern, and plant-based attitude scale) could be latent variables influencing consumer choice.

### Conceptual Framework and Aim of Study

This study was designed to better understand how Brazilian consumers are impacted by different labeling information, particularly regarding the nomenclature used to identify the product, the main ingredient declared, the most used nutritional claims, and the presence and type of the front-of-package labeling (FOPL) system. Given the theoretical expectation that the presence of technical terminology (name) related to the animal-origin product version could mediate choice [26], the following hypothesis was structured:

**Ha:**  *The presence of the nomenclature “Cheese” on the front of the label increases the probability of product choice compared to the terminology prescribed by Brazilian legislation for plant-based analogs (“Plant-based product”).*

The presence of information about soy negatively affects consumers’ perceptions of the quality and sensory value of the product [27,28,29]; therefore, we also investigated whether the presence of information stating “Made with cashew nuts” mediates the choice.

**Hb:**  *The presence of information indicating the main ingredient “Made with cashew nuts” significantly impairs the probability of choosing the product, while the presence of information “Made with milk” increases it.*

Nutritional content claims have been consistently associated with increases in consumer preference [30], and to verify their effect on the choice of muçarela cheese, the following hypothesis was structured:

**Hc:**  *The presence of information indicating the content of calcium, proteins, and vitamins increases the probability of choosing the product.*

Additionally, considering the recent Brazilian legislation that made mandatory the inclusion of FOPL based on the figure of a magnifying glass on food labels containing high levels of sugar, saturated fat, and sodium, and that both products of animal origin and their analogues are subject to FoPL, the following hypothesis was structured:

**Hd:**  *The presence and structure of the FoPL affect product choice probabilities.*

Additionally, there is a theoretical expectation that certain auxiliary dimensions (intrinsic attributes of the subject) may mediate the choice process for plant-based products. Thus, considering that the simulated plant-based product is made with cashew nuts, an ingredient still underexplored in Brazil for the production of analogs, the Food Neophobia Scale [31] was used to understand reluctance to try new foods.

**He:**  *Higher levels of food neophobia are associated with lower probabilities of choosing plant-based products.*

Another dimension that may be relevant in the choice of plant-based foods is the level of environmental concern of individuals. For this, the “Attitude Statements on Climate Change Scale [32]“, which measures attitudes and perceptions regarding climate change and the environmental impact of human activity and eating habits, was employed. The guiding hypothesis for this dimension was:

**Hf:**  *Higher levels of environmental concern increase the probability of choosing the plant-based product.*

To assess whether an individual’s level of health concern was related to the choice process, the Health Consciousness Scale [33,34,35] was used. The use of the HCS is justified by the possible relationship between the choice of plant-based products and the perception of diets that are better for health. The hypothesis that guided this dimension was:

**Hg:**  *Higher levels of health concern increase the probability of choosing the plant-based product.*

Finally, participants’ attitudes towards plant-based products were assessed to verify if greater openness to the benefits of adopting a plant-based diet influenced the choice process of plant-based products. The hypothesis that guided this investigation was:

**Hh:**  *Higher levels of attitudes towards the benefits of adopting a plant-based diet increase the probability of choosing a plant-based product.*

**Figure 1:**
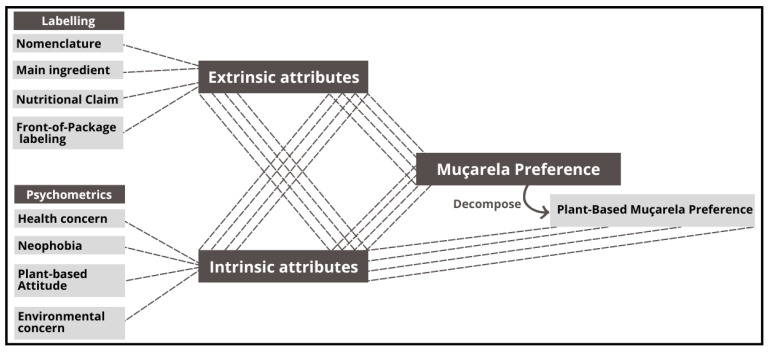
Conceptual framework of the study involving traditional muçarela-style cheese and plant-based alternatives.

In this study, simulated packaging of muçarela—a cheese inspired by Italian mozzarella and the most consumed type in Brazil [36]—was used in both the traditional and plant-based versions (made with cashew nuts). The packaging varied in terms of product nomenclature, type of main ingredient, nutritional claims, and front-of-package labeling (FOPL) structure. Thus, this study investigated the impact of these factors on the consumer choice process. The conceptual framework of the study can be seen in Figure 1.

## 2. Materials and Methods

### 2.1. Data Collection

Two distinct stages of data collection were conducted in this study [37,38], one tracking the eye activity of the respondents (n = 52) and another without eye tracking (n = 509). Both stages were independent and involved different groups of participants. The data collection structure consisted of an electronic questionnaire containing a discrete choice experiment (DCE), which involved various mock packages of traditional and plant-based muçarela cheese, followed by attitudinal questions structured in the form of sub-questionnaires designed to analyze the impact of dimensions associated with the choice process of plant-based cheeses, and finally included sociodemographic questions. The instruments included in these sub-questionnaires covered the Health Consciousness Scale [33], the Food Neophobia Scale [31], the Attitude Statements on Climate Change Scale [39], and the Plant-based Attitude Scale [40].

In the phase involving eye tracking activity, participants were recruited in person at the Department of Economics, Administration, and Sociology of ESALQ/USP, using a convenience sample. For the general questionnaire without eye tracking, recruitment was carried out through social media platforms, using a “snowball” sampling methodology. During the recruitment process, participants were not asked about their cheese consumption, but this information was collected through a specific question in the demographic questionnaire. The study was explained to consumers in the online questionnaire. They were informed that they would participate in the survey using their personal smartphones and that all data would be de-identified and only reported in the aggregate. All participants acknowledged an informed consent statement in order to participate in the study. The project received ethical approval from the Ethics Committee of the Federal Institute of Rio de Janeiro, under registration number 68265023.8.0000.5268. The data collection scheme is shown in Figure 1.

### 2.2. Discrete Choice

**Figure 2:**
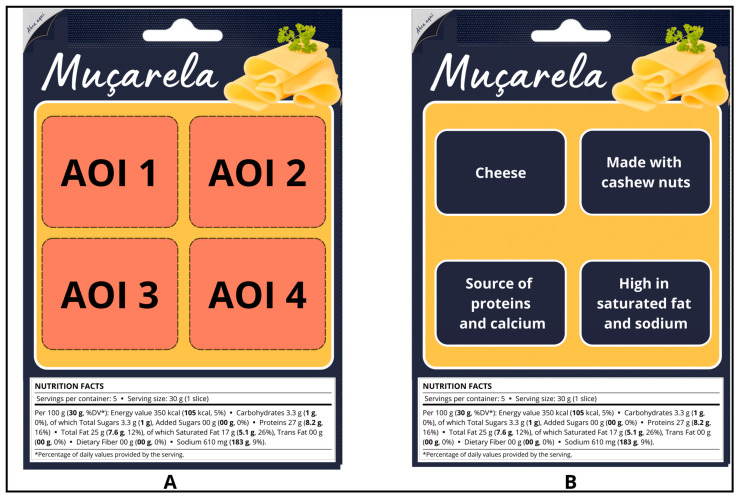
Examples of simulated muçarela cheese packaging used in the discrete choice experiment. Notes: In the left image (**A**), the four areas of interest (AOIs) delimited on the evaluated labels are displayed, while the right image (**B**) shows one of the investigated labels and how the information was distributed within the AOIs.

In the discrete choice experiments, pairs of mock muçarela cheese packages (Figure 2) were presented to participants for them to indicate their preference between the two products presented or if they would opt for none of them. The labels were practically identical, except for the four areas of interest (AOI), where the investigated attributes were evaluated. Figure 2 illustrates an example of evaluated labels and the determined areas of interest. Thus, four attributes, each with three levels, were analyzed in this study (Table 1).

Considering the four attributes evaluated for muçarela cheese, with three different levels for each, a D-optimal matrix [41] was generated to obtain the experimental design to be used in the DCE. Thus, twelve hypothetical products were generated (Table 2).

In both discrete choice experiments, twelve slides with a pair of labels on each were presented to consumers, along with three choice options: “I prefer the product on the left”, “I prefer the product on the right”, or “I do not prefer either of the two products shown”. In all the slides presented, the label on the right differed from the label on the left in terms of the attributes studied. The presentation of the products to consumers followed the design in Table 3. Thus, the first slide displayed labels 1 (left) and 2 (right). Subsequently, the second slide displayed labels 2 (left) and 3 (right), and so forth until the twelfth slide, which displayed labels 12 (left) and 1 (right). This ensured that all labels were shown an equal number of times in both possible positions (right and left)

### 2.3. Modeling Data from the Discrete Choice Experiment

In discrete choice experiments (DCEs), the decision-making process can be analyzed using the theory of random utility, originally proposed by McFadden in 1974. This theory integrates a deterministic model with a statistical structure to describe human choice behavior, recognizing that utility functions contain inherent randomness. Thus, building choice models requires distributive assumptions for the random component [42]. Consequently, the final design of the econometric model depends on two critical decisions: (i) the specification of the utility function (i.e., how the random term enters the conditional Uijt); and (ii) the distributive assumption of the error component [43].

The random utility theory [44] assumes that discrete choice models assume that the utility (*U*) of an individual *i* in choosing alternatives *j*, in choice situation *t* (*Uijt*), can be represented by the following equation:

(1) Uijt=β’i x ijt+εijt

where

*xijt* is a vector of observed variables relative to individual alternatives *j* and *i*;

β’*_i_* is a vector of structural parameters that characterize choices;

ε*_ijt_* is the unobserved error term, which is assumed to be independent of β and x.

Therefore, random utility models can be derived by making various assumptions about the composition and distribution of the unobserved factors *f(εijt)*. In this study, data collected from the discrete choice experiment were structured and analyzed using generalized linear LOGIT models (essentially binary logistic regressions), as opposed to models based on Error Component Random Parameter Logit [45], because for RPL-EC, more than two choices for the individual are required (not just two: choice and non-choice, as is the case in this study). This is because the analysis of the data in this study considered as a choice (Logit value = 1) the fact that all participants in the experiment indicated a choice or non-choice on each slide (i.e., in each discrete choice task). In this model, indicating a preference for one of the two products presented was computed as “choice” (Logit value = 1), while preferring neither of the two products is considered a non-choice (Logit value = 0). The “non-choose” option given to consumers was considered to make the experiment more similar to a real-choice experience, enhancing its ecological validity [46].

In this context, two general empirical models were estimated, i.e., one for the group (n = 52) that performed the DCE with eye tracking activity (DC + ET) and one for the group (n = 509) that responded to the general questionnaire without eye tracking (DC). A third model was derived from the data obtained in the general questionnaire (n = 509), considering only the entries that involved the choice of plant-based muçarela. Plant-based muçarela is an alternative to the traditional product that is not restricted to any consumer niche. That is, the product seeks to replace part of the consumption of the animal-origin version for reasons that go beyond dietary preferences and involve reducing environmental impact and contributing to food security. Thus, it was decided not to restrict data collection to individuals with dietary behaviors more inclined to consume plant-based foods, such as vegetarians, vegans, and flexitarians, but rather to work with a sample of random consumers who declared their preferences for different versions of muçarela.

Therefore, from the general questionnaire (n = 509), two logit models were generated; the first model decomposed the importance of different intrinsic attributes (information present in the areas of interest) and extrinsic attributes (sociodemographic characteristics and attitudes towards health, environment, new foods, and plant-based products) in the choice of muçarela cheeses. To increase the ecological validity of the study, work was carried out on labels and information aligned with products marketed in Brazil, comparing traditional versions of muçarela (animal origin) with plant-based versions. This model aimed to decompose the choice process to assess how the information characterizing the plant-based product impacts consumers when compared to the information and claims of the traditional animal-origin product. A more streamlined second model was obtained from a subsample of individuals who chose some version of the plant-based cheese over the traditional in any of the 12 DCE tasks. For this, the dichotomous choice and non-choice variables were changed to take the value 0 when no product was chosen or some version of animal-origin product was chosen over some plant-origin version. In this second model, to avoid multicollinearity of the intrinsic attributes (since the attributes of the plant-origin product would always be present when Logit was 1), it was decided to remove the variables that exhibited multicollinearity. This resulted in the inclusion of variables more related to extrinsic factors to observe how these factors were related to the choice of the plant-based version of muçarela.

The econometric analysis was performed using a LOGIT (binary) model to estimate the probability of an individual choosing the product (muçarela type cheese) according to the perceptions given by the explanatory variables (attribute levels and variables from the sociodemographic and attitudinal questionnaire) used in each adjusted final model. The model was based on the cumulative statistical (logistic) probability function [47], which can be given by the following equation:

(2) $$P_{i}=\frac{1}{1+e^{-X_{i}\beta }}$$

where *P_i_* represents the probability of occurrence of the product choice event, *X_i_* is a vector of explanatory variables, and *β* is a vector of unknown parameters to be estimated. To estimate the parameters *β*_0, *β*_1…(*β*_n) from the dataset, the maximum likelihood method is used, in which a combination of coefficients is found that maximizes the probability that the sample was chosen [48,49,50]. After estimating the logit model, the marginal effects or partial effects [50] of each attribute were calculated. The calculation of the marginal effect in logistic regression models is essential to providing a clearer and more intuitive interpretation of the results. In non-linear models like logistic regression, marginal effects quantify how small changes in independent variables affect the probability of an event occurring (choosing muçarela), keeping the other variables constant [51]. Because in non-linear models, the estimated coefficient does not faithfully correspond to the probability changes associated with the dependent variable. Thus, unlike a linear regression, in logistic models, the partial derivative of *P*(*Y* = 1) in relation to *X* is not equal to *β* [52]. Thus, the estimated coefficient is more used to understand the direction of change of that variable, where positive and significant values indicate that increases in value in that variable increase the probability of outcome 1 (product choice) and negative and significant values indicate that reductions in that variable reduce the probability of outcome 1 (product choice). For more precise estimates, in LOGIT models, it is customary to calculate the marginal effect of each variable, that is, to obtain the proportion of reduction or increase in the probability of the outcome when a unit of value is changed in the var iable [53].

Greene [54] postulated that much of the econometric analysis seeks to investigate relationships between the covariates, *x*, and the probability of the event, Prob (Y = 1|x) = F(y|x) = F(x’B), which is typically known as partial effects analysis (same marginal effect). In general, through the chain rule,

(3) $$\frac{\partial F\left(y|x\right)}{\partial x}=\frac{\partial F\left(x^{\prime }\beta \right)}{\partial \left(x^{\prime }\beta \right)}\times \beta =f\left(x^{\prime }\beta \right)\times \beta$$

where f() is the density function corresponding to the distribution function, F(). Then, for the logistic distribution,

(4) $$\frac{d\Lambda \left(x^{\prime }\beta_{logit}\right)}{d\left(x^{\prime }\beta_{logit}\right)}=\frac{\exp\left(x^{\prime }\beta_{logit}\right)}{{\left[1+\exp\left(x^{\prime }\beta_{logit}\right)\right]}^{2}}=\Lambda \left(x^{\prime }\beta_{logit}\right)\left[1-\Lambda \left(x^{\prime }\beta_{logit}\right)\right]\;$$

and for the LOGIT model,

(5) $$\frac{\partial F\left(y|x\right)}{\partial x}=\Lambda \left(x^{\prime }\beta_{logit}\right)\left[1-\Lambda \left(x^{\prime }\beta_{logit}\right)\right]\beta_{logit}$$

The values will vary with the values of x so that the set of partial effects is a multiple of the coefficient vector, involving the multiplication of the estimated coefficient β of each explanatory variable by the density function of the logistic distribution. Thus, the partial (marginal) effect is obtained by the partial derivative of the probability curve with respect to the independent variable, keeping the other independent variables constant, resulting in an instantaneous rate of change of the probability curve at that point, which in turn is equivalent to the slope of the tangent line to the curve at that value. This tangent line represents a linear approximation of the probability curve at the selected point, allowing its value to be interpreted as the effect of a unit change in the independent variable on the probability of choice [51].

In the process of constructing the LOGIT model for this study, the “stepwise both” method was used in variable selection and model adjustments [55]. This methodological choice aimed to avoid overfitting, characterized by an overly complex explanation of the data. By applying “stepwise both”, variables were systematically added or removed based on criteria for lower AIC values, allowing the identification of a set of statistically significant variables and avoiding the inclusion of redundant or irrelevant variables. This method provided a balance between the necessary complexity and the desired simplicity, resulting in a model that accurately reflects the trends of the data without overfitting or excessive explanation.

The software used to adjust the model was the R program with the MASS packages for stepAIC, glm for logit, and mfx for marginal effect [56].

### 2.4. Eye Tracking

The biometric reading of eye movements was carried out with the help of an eye tracker (Tobii T120 model) built into a 17-inch monitor with a refresh rate of 60 Hz, a response time of 4 ms, and the ability to capture eye movements at a frequency of 120 Hz (intervals of 8.3 ms). The study used areas of interest to generate statistical representations of the observer’s behavior at strategic locations, associating eye fixation with the metrics analyzed.

The eye tracking metrics employed were total fixation time, i.e., the sum of all periods during which an individual’s eyes remain fixed on the area of interest, and visit count, which corresponds to the total number of times the observer’s eyes are directed and fixed on a particular area of interest.

### 2.5. Attitudinal Scales

In the final phase of the questionnaire, with the aim of capturing information from auxiliary dimensions that may be involved in the choice process of a plant-based cheese alternative, attitudinal scales structured in the seven-point Likert style ranging from “Strongly Disagree” to “Strongly Agree” were used. The choice of specific scales to capture the effect of each dimension (Table 4) was guided by a theoretical rationale that presupposes the existence of a relationship between an individual’s attitudes, their perceptions, and the predisposition to opt for innovative and more sustainably appealing food alternatives.

Firstly, the Health Consciousness Scale, developed by Gould [15], was used to measure individuals’ awareness of their health. This scale is particularly relevant, considering that concern for physical well-being can be a significant motivating factor in the choice of foods perceived as healthier. The Attitudes Towards Climate Change Scale, proposed by De Boer et al. [14], was used to examine the level of concern for the environment and climate change. The inclusion of this scale is based on the premise that environmental awareness can be a determining factor in the preference for more sustainable food alternatives, such as plant-based muçarela cheese.

The third scale adopted was the Food Neophobia Scale, proposed by Ritchey et al. [13] and revised by Rabadán & Bernabéu [57], which aims to measure the reluctance or willingness of individuals to try new foods. This scale is particularly important in the context of introducing innovative food products, as is the case with plant-based muçarela cheese made with cashew nuts. Finally, the Plant-based Attitude Scale, developed by Banovic et al. [19], was used to specifically assess consumers’ attitudes towards plant-based foods, seeking to identify whether there is a favorable predisposition to replace conventional products with plant alternatives.

The original instruments containing the attitudinal scales were translated into Portuguese by a native English-speaking bilingual translator who is fluent in both languages. The translator was assisted by two researchers with extensive experience in the fields of food science and consumer studies. This multidisciplinary collaboration sought to ensure that technical terms and cultural nuances were carefully considered in the translation.

After the translation was completed, a preliminary version of the translated instruments was produced and subjected to a pilot test. This pre-test involved the participation of 30 consumers, selected to appropriately represent the target audience of the research. The aim of this pilot test was to assess the comprehensibility, relevance, and applicability of the scales in the Brazilian context. During this process, minor adjustments were identified and made based on the feedback and responses of the participants, and then the final version of the scales was incorporated into the questionnaire.

#### Analysis of Data from Attitudinal Scales

The attitudinal scales used in this study comprise a series of items and statements that were checked for congruence and factor relations through confirmatory factor analysis [58]. Factorial congruence refers to the extent to which the items of a scale share a common theme and correlate with each other, jointly contributing to the understanding of a specific latent construct or variable. Factorially related items and statements indicate, therefore, that they measure consistent aspects or dimensions of the same underlying phenomenon or concept.

In this study, the scales were selected to capture attitudes related to health (Health Consciousness Scale), environmental concern (Attitude Statements on Climate Change), openness to plant-based products (Plant-based Attitude Scale), and food neophobia (Food Neophobia Scale). The underlying theoretical premises suggest that greater concern for health and the environment, as well as greater openness to plant-based products, may increase the likelihood of choosing plant-based alternatives. Conversely, the theoretical expectation is that higher levels of food neophobia would reduce the likelihood of choosing plant-based products, especially as they are made with a less conventional ingredient, such as cashew nuts.

Once factorial adequacy was checked, the individual mean scores of consumers on each scale were calculated and subsequently entered into the econometric model as continuous variables. This approach allows for quantifying the impact that different levels of these attitudes have on the probability of choosing cheese.

### 2.6. Variables Used in the Models

As previously explained, the dependent variable adopted in this model had a binary structure and was assigned the value “1” when one of the products was chosen and “0” when none of the products were chosen. Reference levels were defined for analysis [59,60], i.e., the attribute levels that would serve as comparison points for the other categories. The “reference category” or “reference level” is the category used as the baseline to compare all other categories of a categorical predictor variable. More precisely, the “reference level” can be considered the value generally observed for that categorical variable and the one that will take a value of zero in the calculation. Thus, the coefficients of the independent variables (non-reference categories) indicate how changes in this variable towards the outcome category are in relation to the reference level.

Therefore, “No Information” was selected as the reference level for the four evaluated attributes, so that all information explored in the Areas of Interest (AOIs) could be compared to the absence of information of that type. The variables included in the models can be seen in Table 5 and Table 6.

## 3. Results

### 3.1. Sociodemographics

The sociodemographic characteristics and estimated frequency of consumption of muçarela and plant-based products of the participant sample are presented in Table 7.

Regarding the metrics related to LOGIT model adequacy, the analysis of the signs observed in the LOGIT coefficients for the variables selected after stepwise indicated good consistency with the theoretical expectations stipulated in the initial hypotheses, such as the increased probability of choosing muçarela cheese when the term “Cheese” is present, the positive effect of health claims related to vitamin, protein, and calcium content, and the negative effect of high sodium and fat content statements, although this effect was more observed in the general sample (n = 509) and not in the group that had eye tracking activity (n = 52). When analyzing the generalized linear LOGIT models in this study, the Nagelkerke pseudo-R squared values vary depending on the type of model applied, reflecting the models’ suitability to the specific context of each analysis. Considering the models with lower AIC values in stepwise, for the general model without eye tracking, a value of 0.2934 was obtained, while for the model that included eye tracking metrics, the value was 0.2836. The model focused exclusively on plant-based cheeses and had a pseudo-R squared of 0.3163.

### 3.2. Confirmatory Factor Analysis

Table 8 presents the metrics used to evaluate the factor structure of the questionnaire, which contains attitudinal questions from the Health Consciousness Scale, Attitude Statements on Climate Change, Food Neophobia Scale, and Plant-based Attitude Scale. Initially, the suitability of the attitudinal data for factor analysis was verified, where a Kaiser–Meyer–Olkin value of 0.860 for the general model containing all sub-questionnaires was found, a value considered adequate for factor analysis adopting the standard cutoff of KMO > 0.6 [61]. Regarding the CFA metrics, a mediocre/satisfactory adjustment of the data was observed if more rigorous cutoff points were considered, including a Comparative Fit Index (CFI) close to 0.95, a Tucker–Lewis Index (TLI) close to 0.95, Standardized Root Mean Square Residual (SRMR) close to 0.08, and Root Mean Square Error of Approximation (RMSEA) close to 0.06 [62]. However, given the heterogeneity of the sub-scales that composed the questionnaire, one focused on health, one on climate change and environmental concern, another related to resistance to new foods, and one on openness to plant-based food, it is possible to safely adopt less rigorous cutoff points as proposed by Hopwood and Donnellan [63], for example, CFI and TLI > 0.90 and RMSEA < 0.10.

### 3.3. Declared Preference in the General Model

The results obtained for the logistic regression of the group without eye tracking are compiled in Table 9. The presence of the term “Cheese” on muçarela labels was associated with a 7.6% increase in the probability of selection as observed in DC and a 15.1% increase in the DC + ET model. Regarding the metrics analyzed in eye tracking, it can be observed that increases in the time of gaze fixation on the term “Cheese” were not related to changes in the probability of choosing the muçarela, while looking longer at the statement “Plant-based product” subtly increased the probability of choice (+2.5%). Regarding the count of visits to the information, it was observed that increases in the number of visits caused reductions in the probability of choice of the muçarela, −3.7% for the information “Cheese” and −1% for “Plant-based product”, suggesting that the need to revisit this information multiple times might be an indication of confusion or insecurity that eventually leads to a lower probability of choosing the products, whether they be the plant-based version or not.

In this study, the presence of the main ingredient information “Made with Milk” (MILK) increased the probability of choosing the muçarela by 6.31%, while such information did not show relevance in the experiment with eye tracking. In the model DC + ET, a reduction of 9.4% was observed in the probability of choosing the product when the information “Made with Cashew Nut” was present.

Claims of nutritional content “Source of vitamins B6 and B12” and “Source of proteins and calcium” on the labels significantly increased the probability of choice by consumers. Specifically, the presence of the vitamin claim increased the probability of choice by approximately 4.97% in the DC and 8.4% in the DC + ET, while the claim of proteins and calcium increased this probability by about 5.69% in the DC and 6.9% in the DC + ET. Finally, the fourth attribute evaluated (high critical nutrient content alert) indicated that “Front-of-pack labelling”, when present, strongly reduced the probability of choosing muçarela in DC, whether in the form of a magnifying glass (−13%) or text (−15.6%).

It has been seen that consumer sociodemographic characteristics and consumption habits exert a significant influence on consumer choices. In DC, when compared to women, men had a −2.7% probability of choosing the muçarela (Table 9). When compared to young people aged between 18 and 25, being between 36 and 60 (−14.2% in the group without eye tracking and −9.5% in the group with eye tracking) and over 60 (−52.4%) strongly reduced the probability of choosing the product (Table 9 and Table 10). When compared to individuals with schooling up to high school, those with higher education or postgraduate studies showed reductions in the probability of choosing the muçarela of −6.4% in DC and −3.7% in DC + ET. In the model without eye tracking, it is observed that individuals in the middle-income range (income between two and six minimum wages) and high income (above six minimum wages) have a significantly lower probability of choosing the products, −9.42% and −4.92%, respectively. The results with eye tracking also reflected this trend, with −14% for middle-income and −10% for high-income.

The frequency of consumption of muçarela and plant-based products positively influenced the probability of choice. Compared to the absence of the habit of consuming muçarela, consuming the product weekly or at least a few times a month increased the probability of choice by 11.7% (Table 9) in DC. The consumption of plant-based foods did not show significance in DC, but in the DC + ET sample, consuming plant-based products a few times a year increased the chance of choosing the muçarela by 8% compared to not having the habit of consuming plant-based foods.

Regarding consumer attitudes, it can be observed in the general model that greater concerns with health and the environment were associated with discrete increases in the probability of choosing the cheese of +1.8% and +1.5%, respectively. Greater openness to plant-based (PB) and levels of neophobia (NEO) were not significant. In the model with eye tracking, only the levels of neophobia were included, so increases in the degree of neophobia represented reductions of −2.1% in the probability of choosing the muçarela.

In the second model obtained from a subsample of individuals who chose some plant-based version of the cheese over the traditional one (Table 11), the impact of the variables related to consumption was maintained, with the frequency of consumption of muçarela a few times a year (CM_YE) being related to a +12.7% probability of choice, consumption of plant-based products a few times a year (CPB_YE) associated with +18.6%, and the frequency of consumption of plant-based products weekly or a few times a month showing a +20.6% increase in the probability of choice. On the other hand, other characteristics were associated with reductions in the probability of choosing plant-based muçarela, such as male gender (−15.4%), age group above 36 and below 60 (−19.1%), higher education or postgraduate level (−4.1%), and middle- and high-income range (−11.4% and −9.3%, respectively). However, interesting differences were observed regarding the marginal effects of the Attitude Statements on Climate Change (ENV) and Plant-based Attitude (PB) scales. It was observed that for the subsample of individuals who made the choice of at least one plant-based version of muçarela over the traditional version, higher levels of concern about climate change and the environment (+5.31%) and greater openness to plant-based diets (Plant-based Attitude Scale) (+5.1%) were related to increases in the probability of choosing the product.

## 4. Discussion

Although a certain balance was observed in the sociodemographic characteristics and consumption habits, the recruitment methods adopted for the DCE and DCE + ET groups may have influenced the profile of participants. This is because online recruitment for the DCE can result in a more diverse sample due to snowball sampling, while convenience sampling used for DCE + ET in a university department may produce a more homogeneous and younger sample, with possible impacts on education, income, and consumption habits [64]. The observed values for Nagelkerke pseudo-R squared, although modest compared to typical linear regression standards, are considered suitable for logistic models, indicating a reasonable fit of the models, where the explained variability is generally more contained due to the binary nature of the dependent variable and the logistic transformation applied [65,66].

The analysis of the attributes investigated was in line with findings from other studies that evaluated plant-based versions of dairy products. Regarding the terminology used to describe the analogous product, Leialohilani and de Boer [67] showed that restricting the use of traditionally established terms for dairy products (e.g., butter, yogurt, and cheese) for commercially designating plant-based products serves as a consumer protection measure. This approach is in line with legislation from various countries, such as the European Union, Brazil, Australia, and the USA [68], which aim to prevent confusion about the origin and composition of products. However, this restriction might also limit consumer perception and the acceptability of plant-based alternatives in the market, where terms like “milk” or “cheese” could facilitate product identification by analogy to dairy equivalents, a strategy suggested by the dairy industry to counter competition from plant-based analogs [69]. This dilemma between protecting consumers and fostering innovation remains a recurrent theme in regulatory discussions, demonstrating the complexity of balancing industry interests and consumer expectations in a rapidly evolving market.

In Brazil, there is currently no definitive regulation for the nomenclature and labeling of plant-based analogs that mimic animal-derived foods, such as “cheese” analogs. The Ministry of Agriculture and Livestock is currently working to establish minimum identity and quality requirements for these products, along with clear guidelines for visual identity and labeling rules [70]. A draft has been prepared and is under public consultation, allowing stakeholders to contribute suggestions. This draft proposes to establish criteria that plant-based analogs must meet to be marketed. However, there are no explicit restrictions in Brazilian legislation on the use of terms that specify the type of cheese, such as “muçarela”, “parmesan”, “cheddar”, etc., as long as these are not preceded or directly associated with the term “Cheese”. This allows manufacturers to explore these nomenclatures on the labels of their plant products, indicating to consumers the type of product the plant food seeks to mimic in terms of flavor, texture, or culinary use.

Although Brazilian legislation prohibits the use of the nomenclature “cheese” in plant products to prevent confusion about the origin of the products, this measure faces practical challenges as consumers accustomed to dairy terms often apply them to plant analogs [67]. Recently, Baptista and Schifferstein [71] observed that animal-related terminology, containers, and claims did not mislead consumers.

Regarding the inspection of consumers’ ocular activity, it was observed that the term “Cheese” is common and widely recognized, allowing consumers to process it quickly without the need for prolonged visual engagement, as they are already familiar with what it represents. In contrast, the term “Plant-based product” represents a more innovative concept and is outside the status quo, which may have required consumers more time for rationalization and understanding of the product, reflecting a need to evaluate how this type of product aligns with their dietary preferences or environmental values. The outcome of this variable suggests that more time spent evaluating this information may favor the choice of plant-based cheese. Teixeira et al. [72] also reported that certain information, when revisited more frequently, shows a reduction in the probability of choice. Some legislation, such as the Brazilian one, is considering recommending declaring the main ingredient of the plant-based product on the label to help consumers have a better understanding of the composition of the products they choose.

The non-significance of the information “Made with Cashew Nuts” (CASW) in the general model can be considered promising for plant-based mozzarella because often the declaration of the plant ingredient, especially soy, tends to reduce the acceptability of plant-based dairy alternatives, whether due to its higher allergenicity, association with GMOs, or consumer prejudices with concerns related to carcinogenesis [73]. Gorman et al. [74] compared a traditional version of ice cream and frozen dairy dessert (based on milk) with plant-based alternatives made with cashew, soy, and coconut. The authors observed that in blind conditions, the taste acceptance scores of the plant-based products were significantly higher than when the ingredient list was informed, while such an effect was not observed in the traditional milk-based product.

The increases in the probability of choosing the product due to the presence of nutritional claims reinforce the importance of nutritional claims on labels to positively influence consumers’ purchasing decisions and reinforce the paths for the enrichment of plant-based products already observed as commercially trending [75]. Different strategies can be explored in this regard. Manufacturers can strengthen the nutritional profile and attraction of their products through focused development, effective labeling, educational campaigns, and obtaining certifications that reinforce the credibility of the products, thus making it possible to positively impact part of the consumers who consider plant-based analogs as highly processed foods and not automatically healthier or more eco-friendly than their animal-origin counterparts [75]. Furthermore, they can explore and enhance the perception of some consumers that plant-based cheese analogs are healthier than normal dairy but that they need improvements in terms of taste and texture [76].

The impact of the statements about the high content of saturated fat and sodium suggests that especially the muçarela have their formulations adjusted to avoid the high content of these nutrients and consequently avoid the presence of the mandatory FOPL. Additionally, the softening of the negative effect when the information is structured with the figure of the magnifying glass may be related to the characteristic of this FOPL structure, as the figure of the magnifying glass may present a certain ambiguity as it incorporates an apparently neutral figure (the magnifying glass) associated with warning statements, such as “High content of saturated fat/added sugars/sodium”, being poorly positioned between being an informative (or reductive) FOPL or interpretive. Reductive FOPLs provide basic information about nutrients without much additional interpretation or judgment of the value of more or less healthy foods. On the other hand, interpretive labels include this information, usually involving visual resources such as colors and figures to help indicate the level of healthiness of the food. Interpretive labels can be nutrient-specific, providing details about individual components, or they can be summary, offering an overall assessment of the nutritional quality. Examples include the multiple traffic light (MTL) system for nutrient-specific labels and the nutri-score for summary labels, while the health star rating (HSR) system combines both elements in a hybrid format [77].

Regarding the findings related to the relationship between sociodemographic profiles and the choice of mozzarella, Alae-Carew et al. [78] reported that women and young individuals aged 24 to 39 years are more likely to report and consume plant-based foods. The review by Szenderák et al. [79] analyzing different profiles of plant-based food consumption reported that in the United States, higher levels of education of individuals were related to a greater preference for plant-based beef analogs and alternatives, while studies from other countries identified a negative and statistically significant relationship between the level of education and the intention to consume plant-based foods in Korea, India, and South Africa.

The relationship between income and the consumption of plant-based alternatives is not yet fully clarified, so such a criterion cannot yet be used as a definitive predictor. This is because the increase in the consumption of plant-based alternatives has been driven by different reasons, including the possibility of including cheaper proteins in the diet when compared to the consumption of animal protein, as well as to meet greater concerns related to environmental issues, health, and social acceptance. Thus, there are studies relating higher income to greater acceptance of plant-based products [79] and studies finding a negative correlation between income and acceptance of plant-based products [80]. Regarding the influence of attitudes on choice, Spendrup & Hovmalm [81] reported that flavor, ease of preparation, health, and the link between diet and climate change are economical ways to increase the choice of plants based on omnivorous consumers.

## 5. Conclusions

The inclusion of the term “Cheese” on muçarela labels significantly influences the product choice probability, which fuels the debate on the role that nomenclature can play in promoting the consumption of plant-based alternatives. The specific designation “Plant-based product” was irrelevant in all models, yet there was a noted need for multiple revisitations of label information in tasks with eye tracking activity, which led to a subtle reduction in choice probability, indicating potential confusion or hesitation. Nutritional claims emerge as positive influences on consumer decision-making, highlighting the potential of strategies that emphasize benefits such as sources of vitamins, minerals, calcium, and protein. The presence of front-of-package labeling (FOPL) declaring a high content of saturated fat and sodium negatively impacted the product choice probability, suggesting that manufacturers pay attention to formulation adjustments so that compulsory labeling is not required. Demographic results indicate significant variations in receptivity to products based on age, gender, income, and consumption frequency, suggesting that social and economic factors play crucial roles in the acceptance of plant-based food alternatives and that communication and marketing efforts be directed accordingly.

## Figures and Tables

**Table 1:** Attributes evaluated in the discrete choice experiment and their respective levels.

Atributes	No. of Levels	Level 1	Level 2	Level 3
Nomenclature	3	Cheese	Plant-based product	No info
Main ingredient	3	Made with milk	Made with cashew nuts	No info
Nutritional claim	3	Source of vitamins B6 and B12	Source of proteins and calcium	No info
Content alert	3	Text–High saturated fat and sodium	Magnifying glass–High saturated fat and sodium	No info

**Table 2:** Optimized profiles used in the discrete choice experiment (D-optimal design).

Profiles	Nomenclature	Main Ingredient	Nutritional Claim	Content Alert
1	No info	Made with cashews nuts	Source of vitamins B6 and B12	Magnifying Glass–FoPL
2	Plant-based product	No info	Source of proteins and calcium	Magnifying Glass–FoPL
3	Plant-based product	No info	Source of vitamins B6 and B12	Text–FoPL
4	No info	No info	No info	Text–FoPL
5	No info	No info	Source of proteins and calcium	No info
6	No info	Made with milk	Source of vitamins B6 and B12	No info
7	Cheese	Made with cashews nuts	Source of proteins and calcium	Text–FoPL
8	No info	Made with milk	Source of proteins and calcium	Text–FoPL
9	No info	No info	No info	Magnifying Glass–FoPL
10	Plant-based product	Made with cashews nuts	No info	No info
11	Cheese	Made with milk	No info	Magnifying Glass–FoPL
12	Cheese	No info	Source of vitamins B6 and B12	No info

Note: FoPL (front-of-package labeling).

**Table 3:** Slide presentation sequence.

Slide	Position		Slide	Position	
	Left	Right		Left	Right
1	Profile 12	Profile 1	7	Profile 6	Profile 7
2	Profile 1	Profile 2	8	Profile 7	Profile 8
3	Profile 2	Profile 3	9	Profile 8	Profile 9
4	Profile 3	Profile 4	10	Profile 9	Profile 10
5	Profile 4	Profile 5	11	Profile 10	Profile 11
6	Profile 5	Profile 6	12	Profile 11	Profile 12

**Table 4:** Attitudinal scales used to capture auxiliary dimensions that may be involved with the choice of traditional and plant-based mozzarella cheese.

Scale	Declarations
Health Consciousness Scale [33]	I reflect on my health a lot.
	I’m very self-conscious about my health.
	I’m generally attentive to my inner feelings about my health.
	I’m constantly examining my health.
	I’m alert to changes in my health.
	I’m usually aware of my health.
	I’m aware of the state of my health as I go through the day.
Attitude Statements on Climate Change [39]	I am very concerned about the environment.
	I would be willing to reduce my consumption to help protect the environment.
	Major political change is necessary to protect the natural environment.
	Anti-pollution laws should be enforced more strongly.
	Major social changes are needed to protect the natural environment.
Food Neophobia Scale * [31]	I do not often try new and different foods.
	I don’t trust new foods.
	If I don’t know what a food is, I won’t try it.
	I do not usually enjoy foods from different cultures.
	Ethnic food looks weird to eat.
	At dinner parties, I do not tend to try new foods.
	I am afraid to eat things I have never had before.
	I am very particular about the foods I eat.
	I won’t eat almost anything.
	I do not generally like to try new ethnic restaurants.
Plant-based Attitude Scale [40]	It is good for the environment to eat more plant-based foods.
	Plant-based food tastes good.
	It is healthy to eat a plant-based diet.
	It is cheap to eat a more plant-based diet.
	I get enough protein if I eat a more plant-based diet.
	I become full from eating plant-based food.
	I would substitute a traditional cheese with a plant-based one if the price were similar.
	I would substitute a traditional cheese with a plant-based one if the taste were similar.

* To maintain consistency in the statements, the reverse sentences of the Food Neophobia Scale (neophilia) were adapted to Neophobia.

**Table 5:** Variables used in the discrete choice experiment model without eye tracking.

Attribute	Definition
NO_INFO *	No information in the AOI regarding the product name, main ingredient, health claim, or high content.
CHES	Binary; “1” if the chosen product contains the information “Cheese”; “0” if not contain.
PLAN	Binary; “1” if the chosen product contains the information “Plant-based product “; “0” if it does not.
MILK	Binary; “1” if the chosen product contains the information “Made with Milk “; “0” if it does not.
CASW	Binary; “1” if the chosen product contains the information “Made with Cashew Nut”; “0” if it does not.
VITB	Binary; “1” if the chosen product contains the information “Vitamins B6 and B12”; “0” if it does not.
CALC	Binary; “1” if the chosen product contains the information “Source of Proteins and Calcium”; “0” if it does not.
MAGN	Binary; “1” if the chosen product contains “FoPL in Magnifying Glass Structure”; “0” if it does not.
TEXT	Binary; “1” if the chosen product contains “FoPL in Simple Text Structure”; “0” if it does not
CM_NO *	Binary; “1” if the subject declares no muçarela consumption, “0” if “CM_WM” or “CM_YE”.
CM_WM	Binary; “1” if the subject declares to consume muçarela weekly/monthly, “0” if “CM_NO” or “CM_YE”.
CM_YE	Binary; “1” if the subject declares to consume muçarela a few times a year, “0” if “CM_NO” or “CM_WM”.
CPB_NO *	Binary; “1” if the subject declares no pant-based consumption; “0” if “CPB_YE” or “CPB_WM”.
CPB_WM	Binary; “1” if the subject declares to consume pant-based weekly/monthly; “0” if “CPB_NO” or “CPB_YE”.
CPB_YE	Binary; “1” if declares to consume pant-based a few times a year, “0” if “CPB_NO” or “CPB_WM”.
SEX_F *	Binary; “1” if the subject declares to be of female biological sex, “0” if “SEX_M” or is not declared.
SEX_M	Binary; “1” if the subject declares to be of male biological sex, and “0” if “SEX_F”.
YOUN *	Binary; “1” indicates age between 18 and 35 years, and “0” if “MATU” or “OLDE”.
MATU	Binary; “1” indicates age between 36 and 60 years, and “0” if “YOUN” or “OLDE”.
OLDE	Binary; “1” indicates age above 60 years, and “0” if “MATU” or “YOUN”.
ES_LOW *	Binary; “1” indicates up to high school, and “0” if “ES_SUP”.
ES_SUP	Binary; “1” when attending college with or without a postgraduate degree, and “0” when “ES_LOW”.
INC_LOW *	Binary; “1” when income is up to two minimum wages, and “0” if “INC_MED” or INC_HIG.
INC_MED	Binary; “1” when it is between two and six minimum wages, and “0” if “INC_LOW” or INC_HIG.
INC_HIG	Binary; “1” when it is above six minimum wages, and “0” if “INC_LOW” or INC_MED.
HCS	Continuous. The average of responses obtained on the Health Consciousness Scale.
ENV	Continuous. The average of responses obtained on Attitude Statements on Climate Change.
NEO	Continuous; the average of responses obtained on the Food Neophobia Scale.
PB	Continuous; the average of responses obtained on the Plant-based Attitude Scale.

* Categories are defined as “reference levels”. Continuous and binary refer to the nature of the variable derived from the attribute in question during tabulation.

**Table 6:** Variables used in the discrete choice experiment model with eye tracking.

Atributes	Definitions
TFD_CHES	Continuous (ms); the total duration of fixations on the AOI with ‘Cheese’ info.
TFD_PLAN	Continuous (ms); the total duration of fixations on the AOI with ‘Plant-based product’ info.
TFD_MILK	Continuous (ms); the total duration of fixations on the AOI with ‘Made with Milk’ info.
TFD_CASW	Continuous (ms); the total duration of fixations on the AOI with ‘Made with Cashew Nut’ info.
TFD_VITB	Continuous (ms); the total duration of fixations on the AOI with ‘Source of Vitamins B6 and B12′ info.
TFD_CALC	Continuous (ms); the total duration of fixations on the AOI with ‘Source of Proteins and Calcium’ info.
TFD_MAGN	Continuous (ms); the total duration of fixations on the AOI with FoPL in the magnifying glass structure.
TFD_TEXT	Continuous (ms); the total duration of fixations on the AOI with FoPL in text structure.
VC_CHES	Discrete; the number of visual inspections on the AOI with ‘Cheese’ info.
VC_PLAN	Discrete; the number of visual inspections on the AOI with ‘Plant-based product’ info.
VC_MILK	Discrete; the number of visual inspections on the AOI with ‘Made with Milk’ info.
VC_CASW	Discrete; the number of visual inspections on the AOI with ‘Made with Cashew Nut’ info.
VC_VITB	Discrete; the number of visual inspections on the AOI with ‘Source of Vitamins B6 and B12′ info.
VC_CALC	Discrete; the number of visual inspections on the AOI with ‘Source of Proteins and Calcium’ info.
VC_MAGN	Discrete; the number of visual inspections on the AOI with FoPL in the magnifying glass structure.
VC_TEXT	Discrete; the number of visual inspections on the AOI with FoPL in text structure.

Notes: FoPL (front-of-pack labeling); AOI (area of interest); ms (milliseconds). Continuous and discrete refer to the nature of the variable derived from the attribute in question during tabulation.

**Table 7:** Sociodemographic characteristics and consumption habits of consumers.

Demographic Characteristics/ Consumption Habits	DC (n = 509)	DC + ET (n = 52)
Biological Sex		
Female	58.15%	67.31%
Male	41.85%	32.97%
Age Group		
Between 18 and 35 years	80.75%	84.62%
Between 36 and 60 years	16.70%	15.38%
Above 60 years	2.55%	0%
Education Level		
Up to High School	22.79%	28.85%
Higher Education and Post-Graduate	77.21%	71.15%
Household Monthly Income		
Up to 2824 BRL	14.15%	11.54%
Between 2824 and 8472 BRL	27.11%	32.69%
Above 8472 BRL	58.74%	55.77%
Mozzarella Cheese Consumption		
Does Not Consume	2.55%	0%
Weekly/Several Times a Month	52.65%	57.69%
Several Times a Year	44.79%	42.31%
Consumption of Plant-based products		
Does Not Consume	65.21%	67.31%
Weekly/Several Times a Month	5.13%	3.85%
Several Times a Year	29.67%	28.85%

Notes: DC is the sample of consumers whose eye activity was not monitored during the discrete choice experiment, while DC + ET is the sample of consumers whose eye activity was monitored during the discrete choice experiment.

**Table 8:** Fit indices observed in the confirmatory factor analysis.

Index	Value
Comparative Fit Index (CFI)	0.924
Tucker–Lewis Index (TLI)	0.918
Root Mean Square Error of Approximation (RMSEA)	0.096
90% Confidence Interval—Lower	0.092
90% Confidence Interval—Upper	0.101
Root Mean-square Residual (SRMR)	0.049
Subscales	KMO
Health Consciousness Scale	
*I reflect on my health a lot*.	0.905
*I’m very self-conscious about my health*.	0.891
*I’m generally attentive to my inner feelings about my health*.	0.84
*I’m constantly examining my health*.	0.926
*I’m alert to changes in my health*.	0.824
*I’m usually aware of my health*.	0.774
*I’m aware of the state of my health as I go through the day*.	0.943
Attitude Statements on Climate Change	
*I am very concerned about the environment.*	0.923
*I would be willing to reduce my consumption to help protect the environment.*	0.9
*Major political change is necessary to protect the natural environment.*	0.808
*Anti-pollution laws should be enforced more strongly.*	0.818
*Major social changes are needed to protect the natural environment.*	0.791
Food Neophobia Scale *	
*I do not often try new and different foods*.	0.893
*I don’t trust new foods*.	0.736
*If I don’t know what a food is, I won’t try it*.	0.761
*I do not usually enjoy foods from different cultures*.	0.76
*Ethnic food looks weird to eat.*	0.67
*At dinner parties, I do not tend to try new foods*.	0.904
*I am afraid to eat things I have never had before.*	0.844
*I am very particular about the foods I eat.*	0.685
*I won’t eat almost anything*.	0.849
*I do not generally like to try new ethnic restaurants*.	0.796
Plant-based Attitude Scale	
*It is good for the environment to eat more plant-based foods*.	0.922
*Plant-based food tastes good*.	0.898
*It is healthy to eat a plant-based diet*.	0.874
*It is cheap to eat a more plant-based diet*.	0.916
*I get enough protein if I eat a more plant-based diet*.	0.877
*I become full from eating plant-based food*.	0.903
*I would substitute a traditional cheese with a plant-based one if the price were similar.*	0.863
*I would substitute a traditional cheese with a plant-based one if the taste were similar.*	0.839
Overall (all scales)	0.86

Note: KMO (Kaiser–Meyer–Olkin test). * To maintain consistency in the statements, the reverse sentences of the Food Neophobia Scale (neophilia) were adapted to Neophobia.

**Table 9:** Estimates of the coefficients of the LOGIT model and respective marginal effect values for discrete choice without eye tracking.

	Logit Model				
Variables	Coefficients	Standard Error	z Value	Pr (>|z|)	Marginal Effects
Intercept	0.662	0.288	2.299	0.0215 **	-
CHES	0.766	0.095	8.087	6.10 × 10^−16^ ***	0.076
MILK	0.620	0.094	6.619	3.61 × 10^−11^ ***	0.0631
CASW	−0.169	0.087	−1.935	0.053	−0.0199
VITB	0.456	0.091	5.022	5.11 × 10^−7^ ***	0.0497
CALC	0.520	0.087	5.969	2.39 × 10^−9^ ***	0.0569
MAGN	−0.974	0.090	−10.853	<2 × 10^−16^ ***	−0.130
TEXT	−1.157	0.091	−12.718	<2 × 10^−16^ ***	−0.156
CM_WM	1.068	0.092	11.586	<2 × 10^−16^ ***	0.117
CPB_YE	−0.297	0.167	−1.778	0.075	−0.0373
CPB_WM	0.201	0.103	1.953	0.051	0.0221
SEX_M	−0.238	0.076	−3.127	0.00176 **	−0.0276
MATU	−0.993	0.095	−10.499	<2 × 10^−16^ ***	−0.142
OLDE	−2.573	0.174	−14.800	<2 × 10^−16^ ***	−0.524
ES_SUP	−0.649	0.104	−6.214	5.15 × 10^−10^ ***	−0.0648
INC_MED	−0.732	0.125	−5.876	4.20 × 10^−9^ ***	−0.0942
INC_HIG	−0.443	0.122	−3.636	0.000277 ***	−0.0492
HCS	0.164	0.033	5.034	4.82 × 10^−7^ ***	0.0187
ENV	0.138	0.042	3.306	0.000946 ***	0.0157
PB	0.043	0.028	1.554	0.120	0.00489

** indicates significance between 1% and 5%, and *** below 1%. Notes: For label information: CHES (Cheese), MILK (Made with Milk), CASW (Made with Cashew Nut), VITB (Source of Vitamins B6 and B12), CALC (Source of Proteins and Calcium), MAGN (FoPL in magnifying glass form), and TEXT (FoPL in text form). For sociodemographic and consumption characteristics: CM_WM (weekly/monthly consumption of muçarela), CPB_YE (plant-based products consumed a few times a year), CPB_WM (weekly/monthly consumption of plant-based products), SEX_M (male sex or not declared), MATU (age between 36 and 60 years), OLDE (age above 60 years), ES_SUP (higher education level), INC_MED (income between two and six minimum wages), INC_HIG (income above six minimum wages). For average scores on attitudinal scales: HCS (Health Consciousness Scale), ENV (attitudes towards climate change), PB (Plant-based Attitude Scale).

**Table 10:** Estimates of the coefficients of the LOGIT model and respective marginal effect values for discrete choice with eye tracking.

	Logit Model				
Variable	Coefficients	Standard Error	z Value	Pr (>|z|)	Marginal Effects
Intercept	5.204	0.939	5.544	2.96 × 10^−8^ ***	-
CHES	4.214	1.433	2.940	0.00328 **	0.151
CASW	−1.173	0.535	−2.191	0.0285 **	−0.094
VITB	1.639	0.438	3.738	0.000185 ***	0.084
CALC	1.280	0.361	3.551	0.000384 ***	0.069
MAGN	−0.679	0.401	−1.693	0.0904	−0.046
TEXT	−0.756	0.469	−1.611	0.107	−0.051
CM_YE	0.445	0.311	1.434	0.151	0.025
CPB_YE	1.775	0.427	4.158	3.21 × 10^−5^ ***	0.080
MATU	−1.151	0.313	−3.680	0.000233 ***	−0.095
ES_SUP	−0.730	0.369	−1.980	0.0478 **	−0.037
INC_MED	−1.736	0.629	−2.761	0.00577 **	−0.140
INC_HIG	−1.757	0.614	−2.862	0.00421 **	−0.100
NEO	−0.352	0.115	−3.066	0.00217 **	−0.021
TFD_CHES	1.054	0.701	1.504	0.133	0.061
TFD_PLAN	0.426	0.147	2.890	0.00386 **	0.025
TFD_VITB	−0.126	0.070	−1.790	0.0734	−0.007
TFD_MAGN	−0.384	0.132	−2.897	0.00376 **	−0.022
TFD_TEXT	−0.126	0.064	−1.959	0.0501	−0.007
VC_CHES	−0.633	0.251	−2.526	0.0115 **	−0.037
VC_PLAN	−0.180	0.067	−2.682	0.00732 **	−0.010
VC_MILK	0.117	0.034	3.431	0.000602 ***	0.007
VC_CASW	0.106	0.057	1.859	0.0631	0.006

** indicates significance between 1% and 5%, and *** below 1%. Notes: For label information: CHES (Cheese), CASW (Made with Cashew Nut), VITB (Source of Vitamins B6 and B12), CALC (Source of Proteins and Calcium), MAGN (FoPL in magnifying glass form), and TEXT (FoPL in text form). For sociodemographic and consumption characteristics: CM_YE (muçarela consumed a few times a year), CPB_YE (plant-based products consumed a few times a year), MATU (age between 36 and 60 years), ES_SUP (higher education level), INC_MED (income between two and six minimum wages), INC_HIG (income above six minimum wages). For average scores on attitudinal scales: NEO (Food Neofobia Scale). For eye tracking variables: TFD_CHES (total fixation duration on ‘Cheese’ information), TFD_PLAN (total fixation duration on ‘Plant-based product’ information), TFD_VITB (total fixation duration on ‘Source of Vitamins B6 and B12′ information), TFD_MAGN (Total duration of fixation on the FoPL magnifying glass), TFD_TEXT (Total duration of fixation on the FoPL in simple text), VC_CHES (number of visual inspections on ‘Cheese’ information), VC_PLAN (number of visual inspections on ‘Plant-based product’ information), VC_MILK (number of visual inspections on ‘Made with Milk’ information), VC_CASW (number of visual inspections on ‘Made with Cashew Nut’ information), VC_VITB (number of visual inspections on ‘Source of Vitamins B6 and B12′ information).

**Table 11:** Estimates of the coefficients of the LOGIT model and respective marginal effect values for discrete choice modeled for specific choice of plant-based cheese.

	Logit Model				
	Coefficients	Standard Error	z Value	Pr (>|z|)	Marginal Effects
Intercept	−1.70214	0.21172	−8.04	9.02 × 10^−16^ ***	-
CM_YE	0.51286	0.07937	6.462	1.03 × 10^−10^ ***	0.1272941
CPB_YE	0.7549	0.09504	7.943	1.97 × 10^−15^ ***	0.1864314
CPB_WM	0.85456	0.17389	4.914	8.91 × 10^−7^ ***	0.2068157
SEX_M	−0.629	0.07737	−8.13	4.30 × 10^−16^ ***	−0.1547892
MATU	−0.805	0.09807	−8.209	2.24 × 10^−16^ ***	−0.191983
OLDE	−0.62569	0.20418	−3.064	0.002181 **	−0.1487731
ESC_SUP	−0.16605	0.08449	−1.965	0.049366 **	−0.0414323
INC_MED	−0.46419	0.10768	−4.311	1.63 × 10^−5^ ***	−0.1140916
INC_HIG	−0.37727	0.10062	−3.75	0.000177 ***	−0.0938544
ENV	0.2134	0.03009	7.091	1.33 × 10^−12^ ***	0.0531535
NEO	−0.04158	0.02727	−1.525	0.127373	−0.0103557
PB	0.20732	0.02666	7.777	7.41 × 10^−15^ ***	0.0516387

** indicates significance between 1% and 5%, and *** below 1%. For sociodemographic and consumption characteristics: CM_YE (muçarela consumption a few times a year), CPB_YE (plant-based products consumed a few times a year), CPB_WM (weekly/monthly consumption of plant-based products), SEX_M (male sex or not declared), MATU (age between 36 and 60 years), OLDE (age above 60 years), ESC_SUP (higher education level), INC_MED (income between two and six minimum wages), INC_HIG (income above six minimum wages). For average scores on attitudinal scales: ENV (attitudes towards climate change), NEO (food neophobia scale), PB (Plant-based Attitude Scale).

## Data Availability

The data presented in this study are available on request from the corresponding author (due to ethical restrictions).
